# Braithwaite’s Retrospect of Practical Medicine and Surgery

**Published:** 1871-09

**Authors:** 


Braithwaite's Retrospect of Practical Medicine and Surgery. Part LXIII. July.

This well-known and valuable semi-annual publication is on our table with its usual punctuality, and filled with its usual valuable collection of materials from the Medical Periodical Literature of Europe.

